# Myriocin Effect on Tvrm4 Retina, an Autosomal Dominant Pattern of Retinitis Pigmentosa

**DOI:** 10.3389/fnins.2020.00372

**Published:** 2020-05-06

**Authors:** Ilaria Piano, Vanessa D’Antongiovanni, Elena Novelli, Martina Biagioni, Michele Dei Cas, Rita Clara Paroni, Riccardo Ghidoni, Enrica Strettoi, Claudia Gargini

**Affiliations:** ^1^Department of Pharmacy, University of Pisa, Pisa, Italy; ^2^Department of Clinical and Experimental Medicine, University of Pisa, Pisa, Italy; ^3^CNR Institute of Neuroscience, Pisa, Italy; ^4^Department of Health Sciences, University of Milan, Milan, Italy; ^5^Aldo Ravelli Center, University of Milan, Milan, Italy

**Keywords:** retinitis pigmentosa, photoreceptor rescue, oxidative stress, Myriocin, mutation-independent approach

## Abstract

Tvrm4 mice, a model of autosomal dominant retinitis pigmentosa (RP), carry a mutation of Rhodopsin gene that can be activated by brief exposure to very intense light. Here, we test the possibility of an anatomical, metabolic, and functional recovery by delivering to degenerating Tvrm4 animals, Myriocin, an inhibitor of ceramide *de novo* synthesis previously shown to effectively slow down retinal degeneration in rd10 mutants (Strettoi et al., 2010; Piano et al., 2013). Different routes and durations of Myriocin administration were attempted by using either single intravitreal (i.v.) or long-term, repeated intraperitoneal (i.p.) injections. The retinal function of treated and control animals was tested by ERG recordings. Retinas from ERG-recorded animals were studied histologically to reveal the extent of photoreceptor death. A correlation was observed between Myriocin administration, lowering of retinal ceramides, and preservation of ERG responses in i.v. injected cases. Noticeably, the i.p. treatment with Myriocin decreased the extension of the retinal-degenerating area, preserved the ERG response, and correlated with decreased levels of biochemical indicators of retinal oxidative damage. The results obtained in this study confirm the efficacy of Myriocin in slowing down retinal degeneration in genetic models of RP independently of the underlying mutation responsible for the disease, likely targeting ceramide-dependent, downstream pathways. Alleviation of retinal oxidative stress upon Myriocin treatment suggests that this molecule, or yet unidentified metabolites, act on cellular detoxification systems supporting cell survival. Altogether, the pharmacological approach chosen here meets the necessary pre-requisites for translation into human therapy to slow down RP.

## Introduction

Recently, several experimental approaches have been proposed, aimed at recovering vision or preventing vision loss caused by retinal degeneration. Photoreceptor degeneration may be initiated by hundreds of different genetic mutations (RetNet, Retinal Information Network)^1^ and visually impaired patients have reached an estimated number of over 20 million worldwide. Among these, individuals with retinitis pigmentosa (RP) develop a typical phenotype characterized by rod degeneration with initial night blindness and loss of peripheral vision. Subsequently, cones also undergo degeneration, jeopardizing daytime vision and visual acuity, eventually leading to legal blindness. RP is a family of disorders typically caused by a single mutation in any of the numerous genes, approximately 70 of which have been identified so far. Thus, defining RP as a highly genetic heterogeneous disease is justified by a large number of underlying mutations, the different functions of the mutated genes, and the variable mode of inheritance (Wright et al., 2010). About 40% of RP cases show autosomal dominant inheritance and 25–30% of these are attributable to mutations in RHO, the gene that codes for rhodopsin, the photosensitive protein of rod photoreceptors.

A biochemical classification introduced in the 1990s (Sung et al., 1991; Sung et al., 1993) identifies two classes of mutations (I and II) on RHO. Class I mutations occur mainly near the C-terminal of the protein, which still retains the ability to bind to 11-*cis*-retinal and to form a functional chromophore but cannot be adequately transported to the outer segment and shows a constitutive activation or an increase in the transducin activation (Mendez et al., 2003). The most common class II mutations occur in the transmembrane or cytoplasmic domains of the protein, resulting in incorrect rhodopsin folding, retention in the endoplasmic reticulum, and inability to bind to 11-*cis*-retinal. Dominant (or dominant-negative) mutations are typically associated with a gain of function of the mutant protein, as observed in the highly studied, highly representative, P23H mutation, which causes retention of rhodopsin at the endoplasmic reticulum (ER) level. The consequent unfolded protein response (UPR) induction and proteosomal inhibition leads to aggregation of high-molecular-weight oligomers, which in turn form toxic intracellular inclusions (Lin et al., 2007; Mendes and Cheetham, 2008; Athanasiou et al., 2018). P23H and similar mutants are not easily approachable through gene therapy, because they require simultaneous suppression of native gene expression and supplementation with a wild-type (wt) version of the gene (MacLaren et al., 2016). Other forms of mutated rhodopsin (T4K, T4N, and T17M) show a lack of glycosylation of the residue N15, which leads to light-dependent retinal degeneration (Tam and Moritz, 2009).

The Tvrm4 mouse is an animal model particularly useful to study RHO mutations and the complex link between primary genetic defect and phenotype manifestation in order to develop appropriate approaches for preserving vision in humans with identical or similar genetic defects. The Tvrm4 strain constitutes an autosomal dominant RP model in which a missense mutation of RHO gene modifies the amino acid 307, isoleucine (ATC), in asparagine (AAC) (Budzynski et al., 2010). Peculiar characteristics of Tvrm4 mice are that they do not express the retinal pathological phenotype and exhibit normal rhodopsin levels if grown in normal housing conditions. However, these mice undergo RP-type retinal degeneration when exposed briefly (between 1 and 5 min) to strong white light (12,000 lux), however harmless to wt controls. The mutation leads to constitutively active mutated opsin (with prolonged dark adaptation times) and exposure to light causes an increase in the production of retinoid metabolites and intermediates of the phototransduction cascade, as well as an increase in mutant-free opsin levels that triggers processes leading to photoreceptor death (Budzynski et al., 2010). Light exposure for 1 to 5 min induces a massive photoreceptor death after 24 h; degeneration is mainly limited to the center of the retina while the peripheral area is less affected (effect probably due to the geometry of the eye) (Gargini et al., 2017). The peculiar features of Tvrm4 mutants, including the possibility of inducing retinal degeneration once neuronal development is completed, make them particularly useful for the study of autosomal dominant RP and retinal remodeling (Stefanov et al., 2019).

Using a widely exploited model of autosomal recessive RP, the rd10 mouse mutant of phosphodiesterase (Chang et al., 2002; Gargini et al., 2007), we have previously shown that long-term topical administration of Myriocin, an inhibitor of the enzyme serine-palmitoyl-CoA transferase involved in the first step of sphingolipids (i.e., ceramides) synthesis, is effective in slowing down the progression of the disease (Strettoi et al., 2010; Piano et al., 2013).

The involvement of sphingolipid metabolism, and in particular ceramide, in RP was first hypothesized in 2004. Tuson and collaborators showed that mutations of the ceramide kinase-like gene (CERKL) were associated with an autosomal recessive RP model (RP26) (Tuson et al., 2004); the role of CERKL in the retinal metabolism of sphingolipids was confirmed later (Garanto et al., 2013).

It is the purpose of the present study to show that Myriocin can be effective in more than one form of RP. To this aim, we administered Myriocin using two distinct protocols (local and systemic administration) immediately after light-induced retinal degeneration in Tvrm4 mice.

The results show that Myriocin is effective in slowing down retinal degeneration in this (fairly aggressive) model of autosomal dominant RP. Surprisingly, Myriocin, administered intraperitoneally for five consecutive days lowers apoptotic processes and increases the anti-oxidant defenses of the retinal cells.

## Materials and Methods

### Animals

Animals were treated in accordance with Italian and European institutional guidelines, following experimental protocols approved by the Italian Ministry of Health (Protocol #14/D-2014, CNR Neuroscience Institute; Protocol #653/2017-PR, Department of Pharmacy, University of Pisa) and by the Ethical Committees of both Institutions. Protocols adhere to the Association for Research in Vision and Ophthalmology (ARVO) statement for the use of animals in research.

Heterozygous (±) Tvrm4 mice (RhoTvrm4/Rho+, from now on “Tvrm4 mice”) with a I307N (near C-terminus) mutation of the rhodopsin gene (*Rho*) mice were used for this study (Budzynski et al., 2010). Tvrm4 mutants are on a C57Bl6/J background and exhibit no retinal phenotype unless exposed to bright light stimuli. Genotyping was performed following Budzynski et al. (2010) and Jackson Laboratory’s indications and only heterozygous mice were used for the experiments.

### Induction of Retinal Degenerating Phenotype and Pharmacological Treatment

In this study we used young, adult mice aged 2–5 months, thus outside the time window of full retinal development (completed at around 30 days of age) and much younger than the manifestation of cellular aging (12 months). The age range chosen was already used in previous studies on Tvrm4 mutants, demonstrating that the light-induced damage is not influenced by the age of the animal (Budzynski et al., 2010; Gargini et al., 2017). Tvrm4 mice were dark adapted for 4 h and then given 1-μl eye drops of 0.5% atropine (Allergan); after 10 min, mice were placed in a black box and exposed to light pulses of 12,000 lux having durations of 2 min (for details, see Gargini et al., 2017). After light induction, the animals were assigned to one of the following experimental protocols:

#### Protocol I: Acute Administration of Myriocin by Intravitreal Injection (i.v.)

Mice were anesthetized with i.p. Avertin (0.5 g/mL 2,2,2-tribromoethanol in ter-amylic alcohol; 20 μl/g body weight). Using a surgical microscope, 1 μl of a 1.88 or 10 mM solution of Myriocin in DMSO was injected into the right vitreous body using a 30-gauge (0.3 × 8 mm) metal needle connected by a plastic tube to a 10-μl glass Hamilton syringe driven by an oil microinjector. The 7-fold dilution, considering the vitreous volume, leads to putative concentration of Myriocin of 0.27 and 1.43 mM, respectively. Left eyes were injected with an equal volume of DMSO. After 24 h, the retinas were isolated from animals under deep anesthesia. Retinas from *n* = 3 mice were fixed in 4% paraformaldehyde (PFA), rinsed in phosphate buffer (PB) 0.1 M, pH 7.4, and stained with ethidium homodimer to reveal cell nuclei. The outer nuclear layer (ONL) was imaged at a Leica TCS-SL confocal microscope using a 568 laser; 12 fields (250 μm × 250 μm) regularly distributed along the retinal surface were imaged and subsequently used to count the nuclei of pycnotic photoreceptors, corresponding to degenerating cells with highly condensed DNA. This method was used as in previous studies (Strettoi et al., 2010, PNAS) as it allows estimating the direct reduction effect of Myriocin on the rate of apoptotic cell death of photoreceptors. The average density of pycnotic cells per retina was calculated and the global average value was established for each experimental group (i.e., Myriocin and corresponding controls). Data were compared statistically using Sigmastat Software. Left and right retinas of *n* = 16 additional mice, injected with 10 mM Myriocin as above, were quickly isolated in cold ACSF and analyzed by HPCL-MS (*n* = 8 mice) and Western blot (WB) assay (*n* = 3) as described below. Animals were killed by cervical dislocation or anesthetic overdose immediately after eye removal.

#### Protocol II: Sub-Chronic Administration of Myriocin by Intraperitoneal Injection (i.p.)

Immediately after light induction, the animals were further subdivided randomly into two groups, a treatment group that received 1 mg/kg/day of Myriocin and a control group that received the vehicle (DMSO). In both cases, the animals were treated for 5 days and, at the end, used for functional analysis (ERG), biochemistry (WB), and immunohistochemistry. At the end of the experimental protocol, the animals were examined to exclude major adverse effects of the treatment (such as the presence of cataract, body weight loss, shaggy fur, or altered sensitivity to anesthesia).

### Electroretinogram (ERG) Recordings

Animals were anesthetized with 20% Urethane (Sigma Aldrich, Milan, Italy), used at a concentration of 0.1 ml/10 g body weight. ERGs were recorded from dark-adapted mice using coiled gold electrodes making contact with the cornea moisturized by a thin layer of gel. Pupils were fully dilated by the application of a drop of 1% atropine (Farmigea, Pisa, Italy). Light stimulation and data analysis were as previously described in detail (Piano et al., 2016). Scotopic ERG recordings were average responses (*n* = 5) to flashes of increasing intensity (1.7 × 10^–5^ to 377 cd^∗^s/m^2^, 0.6 log units steps) presented with an inter-stimulus interval ranging from 20 s for dim flashes to 1 min for the brightest flashes. Isolated cone (photopic) components were obtained by superimposing the test flashes (0.016 to 377 cd^∗^s/m^2^) on a steady background of saturating intensity for rods (30 cd/m^2^), after at least 15 min from background onset. The amplitude of the a-wave was measured at 7 ms after the onset of the light stimulus and the b-wave was measured from the peak of the a-wave to the peak of the b-wave.

The leading edge of the a-wave was fitted to the model of the activation phase of the rod G-protein transduction cascade (Lamb and Pugh, 1992) according to the following equation:

(1) F(t)=exp[-1/2ΦA(t-t)e⁢f⁢f]2

where *F*(*t*) is the fraction of the circulating current normalized to its dark value, *A* is the amplification factor with the units of s^–2^/Φ, expressed in terms of the gain parameters of the cascade stages, and *t*_*eff*_ is inclusive of any delay contained in both the response and the instrumentation. Further details for the application in the RP animal model are given in Gargini et al. (2007).

Oscillatory potentials (OPs) were also measured in both scotopic and photopic conditions. OPs were extracted digitally by using a fifth-order Butterworth filter as previously described (Hancock and Kraft, 2004; Lei et al., 2006). The peak amplitude of each OP (OP1–OP4) was measured. The ERG data for each condition of light induction were collected from at least six different animals.

### Western Blot

Retinas from Tvrm4 mice were lysed in modified RIPA buffer as described before (Piano et al., 2019) and protein was quantified with the Bradford assay (Bio-Rad). For each experiment performed, three different samples (each one composed of both retinas mixed together) for each experimental group were loaded on the gel, each from an animal previously used for ERG recordings. Proteins (25 μg) were separated onto a pre-cast 4–20% polyacrylamide gel (Mini-PROTEAN^®^ TGX gel, BioRad) and transferred to PVDF membranes (*Trans*-Blot^®^ Turbo^TM^ PVDF Transfer packs, Bio-Rad). Membranes were blocked with 5% of non-fat dry milk (Bio-Rad) diluted in Tris-buffered saline (TBS, 20 mM Tris–HCl, pH 7.5, 150 mM NaCl) with 0.1% Tween 20. Primary antibodies against rhodopsin (cod. R5403, antibody concentration: ∼1 mg/ml, working dilution1:1000, Sigma Aldrich), cone-opsin blue and cone-opsin red/green incubated altogether (cod. AB5407 and AB5405, respectively antibodies concentration 1 mg/ml, working dilution 1:500, Millipore), caspase-3 (cod. sc-7272, antibody concentration: 200 μg/ml, working dilution 1:100, Santa Cruz Biotechnology), and Sod1 (cod. SAB5200083, antibody concentration 1 mg/ml, working dilution 1:500, Sigma Aldrich) were incubated overnight at 4°C. After performing three washings for 10 min each in T-TBS (TBS buffer with 0.1% of Tween-20), membranes were incubated with secondary antibodies (anti-mouse or anti-rabbit HRP conjugated, cod. AP308P or AP147P, respectively, antibodies concentration 1 mg/ml, working dilution 1:5000; Sigma Aldrich) for 2 h at room temperature. The immunoblot signal was visualized by using an enhanced chemiluminescence substrate detection system (Luminata^TM^ Forte Western HRP Substrate, Millipore). The chemiluminescent images were acquired by Chemidoc XRS+ (Bio-Rad). Densitometry was undertaken using Bio-Rad ImageLab software.

Each protein of interest for the study was normalized for total protein content by using the Stain Free Technology (Bio-Rad) (Gürtler et al., 2013). To optimize the number of animals and the use of samples obtained, in some cases different proteins were analyzed on the same membrane following a stripping procedure preceded by three washings (10 min each) in glycine buffer, pH 2, followed by three washings in T-TBS and blocking in 5% of non-fat dry milk. The incubation of primary and secondary antibodies was performed with the same protocol described above.

### Tissue Preparation, Histology, and Immunocytochemistry (IHC)

For retinal histological studies, mice were deeply anesthetized with intraperitoneal injections of 0.1 ml/5 g body weight Avertin as described above, their eyes quickly enucleated, and the animals killed by cervical dislocation. Eyes were labeled on the dorsal pole, the anterior segments were removed to obtain eye cups and fixed in 4% paraformaldehyde (PFA) in 0.1 M phosphate buffer, (PB), pH 7.4, for 30 min or 1 h, at room temperature. Eye cups were washed extensively in PB, infiltrated in 30% sucrose in PB at 4°C, frozen in Tissue-Tek O.C.T. compound (4583, Sakura Olympus, Italy) using cold isopentane and stored at −80°C until use. The eyes were used to prepare retinal whole mounts, in which the retina was separated from the pigment epithelium and flattened by making four radial cuts toward the head of the optic nerve, maintaining a reference on the dorsal pole. Areas of maximum photoreceptor loss were imaged on retinal whole mounts stained with 0.2 mM ethidium homodimer (Strettoi et al., 2010) and mounted with the photoreceptor side facing the coverslip. IHC on whole mounts was performed following Barone et al. (2012), by incubation in (a) block solution with 0.3% Triton X-100, 5% goat serum in 0.01 M phosphate buffer saline (PBS); (b) anti cone-arrestin (cod. AB15282, 1 mg/ml; working dilution 1:1000) primary antibody, overnight at 4°C; and (c) anti-rabbit 568-conjugated secondary antibody (cod. SAB4600310, 2 mg/ml, working dilution 1:5000). After rinsing in PBS, specimens were mounted in Vectashield (H-1000; Vector Laboratories, Burlingame, CA, United States) and coverslipped.

Ethidium-stained retinal whole mounts were examined with a Leica TCS SL confocal microscope equipped with a 568-nm laser using a 40×/1.25 oil objective. ONL series (*n* = 10 images along the *z* axis, encompassing the outermost 10 μm) and having a size of 250 × 250 μm were acquired for each retinal samples along the dorsoventral and peripheral axis (*n* = 12 fields/retina), using identical acquisition parameters for treated and control samples. Pycnotic profiles (corresponding to actively dying photoreceptors) were counted on projection images of each confocal series using the manual object counting tool of Metamorph. Counts were averaged and statistically compared using SigmaStat software.

Low-magnification images of retinal whole mount for cone-arrestin preparations were obtained with the Nikon Ni-E microscope using CFI plan fluor 10×/0.3 NA objective, with 16 mm working distance; images were tiled with Nis “Tiles and Positions” software to reconstruct the entire retinal surface. Areas of maximum photoreceptor loss were measured on full retinal montages saved as tiff files and transferred to an ImageJ analyzer. Images were homogeneously thresholded for brightness and the darkest, central region surrounding the optic nerve head (ONH) was automatically tracked and measured. This method was chosen as, at advanced stages of degeneration, cone-arrestin staining highlights clearly the dark, central area devoid of cones, allowing a quick and reproducible measurement of the extension of this zone in treated and control cases. This zone coincides with the central part of the retina where pycnotic (dying) photoreceptors are visible at early stages.

### Ceramide Content

The third group of retinas from light-induced Tvrm4 mice, injected intravitreally with 10 mM Myriocin, as described in protocol 1, was used for paired ERG recordings and biochemical assessment of retinal ceramides. Freshly isolated retinas were rinsed in ACSF, placed in Eppendorf tubes and weighed on an analytical balance. The entire procedure took less than 2 min; samples were then snap frozen on dry ice stored at −80°C until use. Animals were killed by cervical dislocation immediately after eye removal.

The analysis and the extraction procedure from the retina sample for the determination of sphingolipids were carried out as already described (Platania et al., 2019). Briefly, retinas were homogenized in the TissueLyser (Qiagen, Hilden, Germany) and then aliquoted for the extraction of sphingolipids. Sphingolipids were extracted using a mixture of water/methanol/chloroform (1:6:3, v/v/v). The analytical system consisted of a UPLC Dionex 3000 UltiMate (Thermo Fisher Scientific, United States) connected to an ABSciex 3200 QTRAP with electrospray ionization TurboIonSprayTM source (AB Sciex S.r.l., Milan, Italy). Quantitative sphingolipid determination was performed by multiple reaction monitoring (MRM) using as internal standard C12 ceramide. The separation was carried out on a reversed-phase BEH C-8 10 × 2.1 mm, 1.7 μm particle size (Waters, Milford, MA, United States) by mixing eluent A (water + 2 mM ammonium formate + 0.2% formic acid) and eluent B (methanol + 1 mM ammonium formate + 0.2% formic acid).

### Statistical Analysis

Statistical comparisons for ERG and WB analysis were performed with analysis of variance (ANOVA) one- or two-way test followed by Bonferroni-corrected *t* test using Origin Lab 8.0 software (Microcal, Northampton, MA, United States).

## Results

Two studies previously published by the authors (Strettoi et al., 2010; Piano et al., 2013) showed that ocular administration of Myriocin, through instillation of nanosphere-based eye drops, was effective in slowing down the primary degeneration of rods as well as the degeneration of cones in rd10 mutants, mimicking typical, autosomal recessive RP. Current literature correlates the mechanism of action of Myriocin to the reduction of long-chain ceramides, mediated by the inhibition of the *de novo* synthesis of these important bioactive sphingolipids (Sanvicens and Cotter, 2006; German et al., 2006; Ranty et al., 2009; Kurek et al., 2017).

**FIGURE 1:**
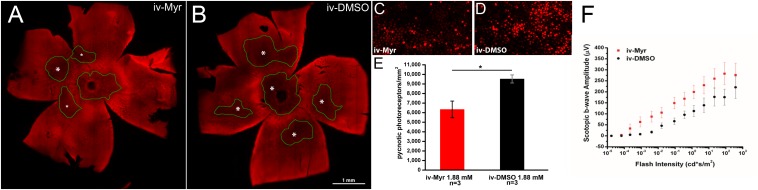
Myriocin intravitreal injections in Tvrm4 mice. **(A,B)** Montages of images of whole-mount retinas stained with ethidium nuclear dye (red fluorescence). The focal plane is on the outer nuclear layer (ONL). Darker zones (green-encircled) indicate areas where photoreceptors have degenerated and their nuclei are missing. Larger degenerating zones are labeled with larger asterisks (*). The Myriocin-injected side shows dark zones that are visibly smaller (labeled with smaller asterisks) and less numerous compared to the control. **(C,D)** High magnification of the ONL of retinal whole mounts as those shown in A and B. Bright red nuclei belong to photoreceptors that entered in a stage of DNA condensation (pyknotic nuclei), visibly less numerous in the Myriocin-injected compared to the control eye. Imaged fields have consistent eccentricity and have been acquired with identical confocal microscope settings. **(E)** In the underlying bar graph, the average number of pyknotic nuclei is compared in retinas treated with Myriocin (red bars) and retinas treated with vehicle (black bars). Measurements are from three different mice (*n* = 3). **(F)** Sensitivity curve of the scotopic b-wave amplitude in Myriocin-treated (red squares) and vehicle-treated eyes (black circles) (*n* = 3). Bar values represent mean ± SEM.

To verify whether this therapeutic approach can be considered mutation-independent, we administered Myriocin to the Tvrm4 mouse, which carries a different pathogenic mutation, with a different transmission modality compared to the rd10 mutant. Pilot experiments were done following the protocol previously used on rd10 mutants and based on a single intraocular administration of Myriocin, as this route was proven by us to produce readily-measurable retinal effects. Two-month aged animals were exposed to a strong white light for 2 min to trigger retinal degeneration; after 24 h, Myriocin was injected in the vitreous body of the right eye. The left eye, injected with vehicle, served as control. Figure 1 illustrates whole-mount retinas stained with ethidium and imaged at the ONL (Figures 1A,B): the dark areas are zones of decreased fluorescence corresponding to thinning of the ONL and of lost missing photoreceptors. The extension of degenerated zones (circled in green) is visibly reduced in the retina treated with Myriocin with respect to the control side. Coherently, the number of pycnotic profiles (corresponding to condensed nuclei of actively dying photoreceptors) illustrated in Figures 1C,D is lower in retinas treated with Myriocin than in controls. The bar graph in Figure 1E shows an average decrement of over 1/3 dying photoreceptors in the retina of Myriocin-injected mice. As for the rd10 mutants used in the previous study (Strettoi et al., 2010), also in acutely treated Tvrm4 animals, the retinal function was tested by recording the ERG response. The experimental group shows a collective trend toward a higher conservation of the amplitude of the scotopic b-wave in Myriocin-injected mice, however, the difference with controls reaches statistical significance (Figure 1F).

**FIGURE 2:**
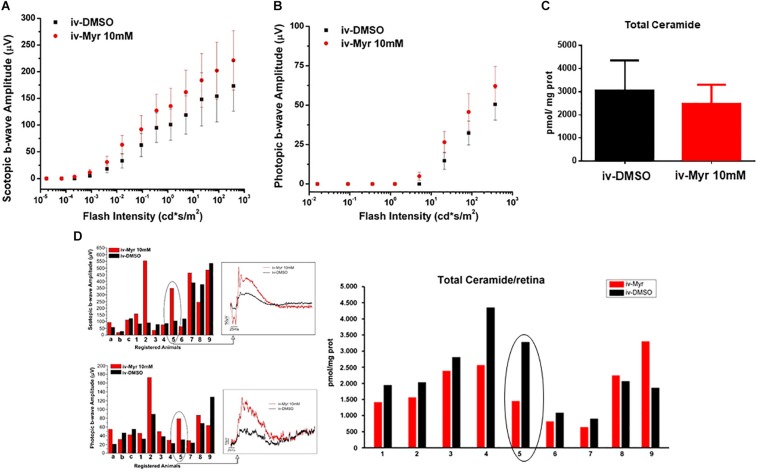
Correlation between the decrease of ceramide content and functional recovery. **(A,B)** Sensitivity curve, respectively, of scotopic and photopic b-wave amplitude as a function of light flashes of increasing intensity. Although not significantly, the amplitude of the ERG response is increased in the eyes injected with Myriocin (red dots) compared to the eyes treated with the vehicle (black dots). Values represent mean ± SEM (*n* = 12; 11). **(C)** Bar graph showing quantification of Ceramide content in the same retinal samples used for ERG analysis. The value represents mean ± SEM (*n* = 9). **(D)** Correlation between retinal ceramide levels and functional activity. The bar graph on the left shows the scotopic and photopic b-wave amplitude recorded from Tvrm4 mice injected with 10 μM Myriocin in the right eye (red bars) and with vehicle in the left eye (black bars); representative (i.e., average-size) traces recorded at the highest luminance used (377 cd*s/m^2^) are shown in the squared boxes on the right. The bar charts in the right of the panel show HPLC-MS quantification of ceramide content in the same retinas used for the ERG analysis. A correlation between the functionality of retinal neurons and ceramide levels is noted, even in cases when Myriocin does not show a protective effect: for instance, animal 5 has a larger ERG response of the eye treated with Myriocin, which corresponds to reduced levels of ceramide; animal 9 shows a smaller amplitude of the ERG in the Myriocin-treated eye, which, however has a higher ceramide content, possibly as a result of improper eye injection procedures.

To increase the chances of retinal rescue, we tested the effects of a single intraocular injection of 10 mM Myriocin by measuring in the same mice both retinal function by ERG recordings and total ceramide levels assessed by HPL-MS. Figures 2A,B illustrate the sensitivity curve of scotopic and photopic ERG, respectively, recorded from animals in which the right eye was injected with 10 mM Myriocin (red dots) and the left eye with vehicle (black dots); Figure 2C shows retinal ceramide levels measured in the same animals. Effective recovery of a retinal function cannot be demonstrated (mainly due to the high variability encountered). Yet, an inverse correlation between the preservation of retinal light-responses and retinal ceramide content is observable (Figure 2D), albeit the finding does not reach statistical significance. The analysis of individual animals shows that when the amplitude of the ERG response in the eye treated with Myriocin is higher than in the eye injected with DMSO, ceramide levels are lower in Myriocin-treated eye than in the vehicle-treated one (sample no. 5). On the other hand, sample no. 9, in which the treatment was not effective, shows that the amplitude of the ERG is lower in the eye with higher ceramide levels. These data, showing a high individual variability, nevertheless indicate a direct correlation of retinal ceramide levels and degeneration and death of photoreceptors in Tvrm4 mutants (Figure 2D).

To test the possible therapeutic efficacy of Myriocin on a longer time scale, and considering (a) the impossibility to use repeated ocular administrations in mice and (b) the relatively quick rate of central retinal degeneration in Tvrm4 mutants (Gargini et al., 2017), we delivered Myriocin by intraperitoneal (i.p.) injections at the dose of 1 mg/kg/day for 5 days. The dose, route, and protocol of administration were based on existing literature (Glaros et al., 2007; Glaros et al., 2010; Lee et al., 2012).

**FIGURE 3:**
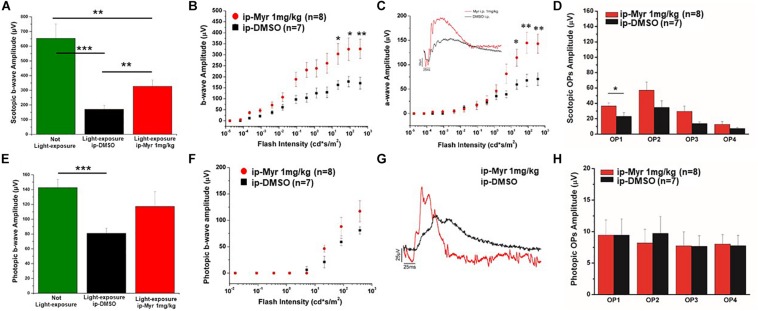
Functional recovery in Tvrm4 mice after Myriocin intraperitoneal (i.p.) treatment. **(A)** The bar graph shows the scotopic b-wave amplitude measured at the highest luminance in non-induced control animals (green bar), in animals treated, after light induction, with vehicle (black bar), or with Myriocin (red bar). **(B)** The sensitivity curve of b-wave amplitude as a function of luminance shows a significant increase in Myriocin-treated versus control group. **(C)** The sensitivity curve of a-wave amplitude as a function of luminance shows a significant increase in Myriocin-treated versus control group. The inset represents an example of ERG traces recorded at the highest luminance used (377 cd*s/m^2^). **(D)** Scotopic OPs obtained from ERG traces; the bar graph shows that only the OP1 relative to photoreceptor activity is significantly larger in Myriocin-treated mice with respect to the control mice. **(E)** Photopic ERG recordings from the not light-exposed Tvrm4 mice (green bar), vehicle-treated (black bar), and Myriocin-treated (red bar) mice. **(F)** The sensitivity curve of b-wave amplitude as a function of luminance shows a tendency to be larger in the Myriocin-treated group with respect to the control group. **(G)** Representative example of ERG traces recorded at the highest luminance used (377 cd*s/m^2^) superimposed on a steady background (30 cd/m^2^). **(H)** Photopic OPs obtained from ERG traces. The value represents mean ± SEM; *p ≤ 0.05, **p ≤ 0.01, ***p ≤ 0.001.

The results obtained by recording the ERG in Tvrm4 animals exposed to light and treated via i.p. with Myriocin or vehicle, for 5 days, are shown in Figure 3. The bar graph shown in A compares the amplitude of the scotopic b-wave obtained from Tvrm4 animals where the degeneration was not induced (green bar—healthy control) with the two groups of animals exposed to light (pathological phenotype) treated with the vehicle (black bar) and with Myriocin (red bar). Figures A–C show that the treatment with Myriocin is effective in reducing retinal damage compared to animals treated with the vehicle alone, although photoreceptor preservation is not complete (Ctr vs. DMSO *p* = 0.0008; Ctr vs. Myr *p* = 0.006). Figure 3B shows that the amplitude of the b-wave is significantly larger (*p* = 0.02112; *p* = 0.02744; *p* = 0.01, respectively) in the group of animals treated with Myriocin (*n* = 8, red dots) compared to the group of animals treated with the vehicle (*n* = 7, black dots). Similarly, Figure 3C shows that also the amplitude of the a-wave is significantly higher in the animals treated with Myriocin with respect to the vehicle group (*p* = 0.018; *p* = 0.0093; *p* = 0.01, respectively); this effect is also confirmed by the a-wave analysis according to Lamb and Pugh (1992). The amplification parameter (A, s^–2^/Φ) and the effective delay of the responses (*t*_*eff*_) are shown in Tables 1, 2. It can be observed that the amplification factor is significantly reduced and that the total delay is substantially increased in the group treated with the vehicle alone with respect to the Myriocin-treated group and also with respect to the healthy control; the analysis also shows that the injection of Myriocin is effective in restoring the A parameter near the physiological condition. Figure 3D shows that the OP amplitude shows a significant increase, mainly in the OP1 (directly correlated with the function of photoreceptors) in Myriocin-treated mice with respect to the vehicle group (*p* = 0.048).

**TABLE 1:** Average value of amplification factor (A) of the a-wave measured at 14000. photoisomerization*rod^–1^ (Φ).

**Experimental Group**	**A (s**^–2^/Φ) **Mean** ± **St. Err.**	***P* value (calculated respect to ip-DMSO)**	***P* value (calculated respect to No light-exposure)**
ip-DMSO	4.258 ± 0.45056		
ip- Myr	9.56067 ± 1.13184	0.0066**	N.S. (*P* > 0.05)
No light-exposure	9.7215 ± 0.97015	0.0092**	

**TABLE 2:** Average value of effective delay (Teff) of the a-wave measured at 14000 photoisomerization*rod^–1^ (Φ).

**Experimental Group**	**Teff (s) Mean** ± **St. Err.**	***P* value (calculated respect to ip-DMSO)**	***P* value (calculated respect to No light-exposure)**
ip-DMSO	0.01097 ± 3.61E-4		
ip- Myr	0.00996 ± 2.27E-4	0.036*	0.00001***
No light-exposure	0.00772 ± 1.42E-4	0.00007***	

Photopic ERG recordings are illustrated in Figures 3E–H; massive degeneration of the cones in the Tvrm4 mutant results in a significant reduction of the photopic b-wave amplitude with respect to the healthy control mice (Ctr vs. DMSO; *p* = 0.00054). The treatment with Myriocin shows a tendency, although not significant, to preserve the amplitude of the b-wave due to the activation of cone photoreceptors. The analysis of photopic OPs shows no differences between the two groups of mice.

**FIGURE 4:**
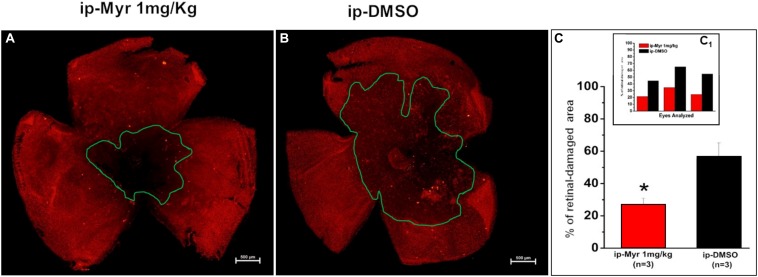
Reduction of the photoreceptor-degeneration area after i.p. administration of Myriocin. **(A,B)** Representative images of retinal whole mounts obtained from control and Myriocin-treated mice obtained by anti-cone arrestin immunostaining (red fluorescence). The green lines surround the area affected upon the induction of the mutation by light exposure. **(C)** Quantification of the affected area calculated as a percentage of the total retinal area from three different experiments (shown in the insert bar graph, **C1**), showing a significant reduction in animals treated with Myriocin (red bars) compared to animals treated with vehicle (black bars). The values reported in the main bar graph represent global averages of all the experiments; Values are shown as mean ± SEM (*n* = 3, for each analyzed group); *p ≤ 0.05.

The retinas obtained from the animals used for electrophysiological recordings were processed for immunohistochemistry and biochemistry experiments. Figures 4A,B show a retina obtained from an animal treated with Myriocin and one treated with the vehicle stained with cone-arrestin antibodies; the central area of degeneration (highlighted by the green outline) is largely reduced in the retina from the animal treated with Myriocin with respect to the control. Measurement of the degeneration zone with respect to the total retinal area, reported as a percentage, is shown in the bar graph of Figure 4C, proving how systemic administration of Myriocin for 5 days significantly reduces (*p* = 0.03471) the area of photoreceptor death. These data also indicate the ability of Myriocin or of an active metabolite to cross the blood–retinal barrier and reach the retina, which is known to weaken in neurodegenerations, thus allowing the passage of molecules useful for therapeutic treatment (Acharya et al., 2017).

**FIGURE 5:**
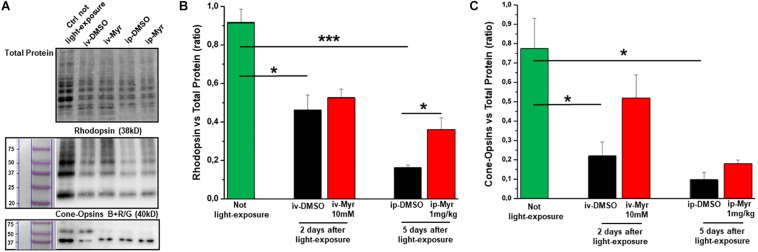
Preservation of rhodopsin and cone-opsins level following treatment with Myriocin. **(A)** Representative example of WB experiment of rhodopsin and cone-opsins content. **(B)** Quantification by optical densitometry of rhodopsin levels in Tvrm4 animals not exposed (green bar) and treated with Myriocin (red bar) or with vehicle (black bar) by both intravitreal and intraperitoneal injection after light induction. The bar graph shows that the levels of rhodopsin are significantly reduced in the animals treated with DMSO with respect to the non-exposed controls; i.p. treatment with Myriocin results in a rhodopsin content significantly higher with respect to the group of animals treated with vehicle. **(C)** Quantification by optical densitometry of cone-opsins levels in Tvrm4 animals not exposed (green bar) and treated with Myriocin (red bar) or with vehicle (black bar) by both intravitreal and intraperitoneal injection after light induction. The bar graph shows that the levels of cone-opsins are significantly reduced in the animals treated with DMSO with respect to the non-exposed control. Treatment with Myriocin, by both routes of administration, leads to an increase in cone-opsins levels, although not statistically significant. Values are shown as mean ± SEM (*n* = 3, for each analyzed group); *p ≤ 0.05, ***p ≤ 0.001.

In order to confirm the rescue effects described above, we measured the levels of rhodopsin and of blue and red/green cone-opsins by WB analysis (Figures 5A–C). The bar graph in Figure 5B shows that after induction of the mutation by light exposure, rhodopsin levels are significantly reduced compared to the levels measured in a healthy control animal (Ctr vs. i.v.-DMSO *p* = 0.012; Ctr vs. i.p.-DMSO *p* = 0.0004). The graph also shows that previously described i.v. treatment with Myriocin is not sufficient to preserve a number of rods such as to result in a significant increase in rhodopsin content; on the other hand, i.p. treatment with Myriocin is effective in saving a larger number of photoreceptors, resulting in a significant increase of this protein (i.p.-DMSO vs. i.p.-Myriocin *p* = 0.035). Figure 5C shows a similar trend also for the cone-opsins levels, which are significantly reduced in the induced animals treated with the vehicle alone with respect to healthy controls (Ctr vs. i.v.-DMSO *p* = 0.033; Ctr vs. i.p.-DMSO *p* = 0.014). Again, although not significantly, the treatment with Myriocin shows a trend toward cone preservation as shown in the bar graph of Figure 5C. These data are in agreement with ERG recordings that show a significant recovery only for the scotopic protocol.

**FIGURE 6:**
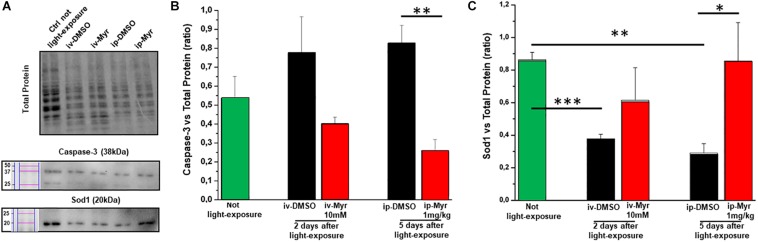
Systemic Myriocin treatment increases the level of Sod1 protein. **(A)** Representative example of WB experiment of caspase-3 and Sod-1 content. **(B)** Quantification by optical densitometry of caspase-3 levels in Tvrm4 animals treated with Myriocin (red bar) or with vehicle (black bar) compared to the level in non-exposed mice (green bar). The bar graph shows that the levels of the pro-apoptotic marker caspase-3 are reduced in the group of animals treated with Myriocin. **(C)** Quantification by optical densitometry of Sod1 levels in Tvrm4 animals not light-induced (green bar), treated with vehicle (black bar), or treated with Myriocin (red bar). The bar graph shows that the levels of the anti-oxidant enzyme Sod1 are significantly increased in the group of mice treated with Myriocin, with respect to both the group of non-degenerate control animals and the group of mice treated with the vehicle alone. Values are shown as mean ± SEM (*n* = 4, for each analyzed group); ^∗^*p* ≤ 0.05, **p ≤ 0.01, ***p ≤ 0.001. **(B)** Representative example of WB experiment.

The levels of caspase-3, a specific marker of apoptosis, were also measured by WB assay (Figure 6A), to confirm the reduction of common apoptotic pathway activation. Figure 6B shows how the levels of caspase-3 are increased in degenerating retina with respect to the control healthy group. Furthermore, i.p. treatment with Myriocin significantly reduces (*p* = 0.0057) the levels of caspase-3 compared to those obtained from control animals (*n* = 4/experimental group). This result confirms the anti-apoptotic effect of Myriocin, at the retinal level, also when administered systemically.

Finally, to assess whether Myriocin (or any derivative metabolites) could act via signaling pathways known to act in inherited retinal degeneration, we analyzed retinal protein levels of Sod1, one of the main enzymes of the physiological anti-oxidant system. Figure 6C shows how, in retinas obtained from light-induced Tvrm4 mice, the Sod1 content is significantly reduced with respect to the control healthy mice (Ctr vs. i.v.-DMSO; *p* = 0.0008; Ctr vs. i.p.-DMSO; *p* = 0.008). This result indicates a reduced ability of the damaged photoreceptors to remove ROS from the cellular environment. In both treatment protocols (i.v. and i.p.) with Myriocin, it is possible to note an increase in Sod1 levels toward physiological ones, although only in the case of i.p. administration is this increase significant (*p* = 0.035).

These results indicate that Myriocin, administered i.p., directly or by means of yet unknown metabolic derivatives, reaches the interior of the eye and exerts a protective effect on RP-like retinal degeneration. Besides the known activity as a suppressor of ceramide *de novo* synthesis and an anti-apoptotic agent, Myriocin could act, in medium and long-term treatments, on other cellular pathways, including induction of detoxifying anti-oxidant systems.

## Discussion

Previous studies have shown that increased ceramide levels in the rd10 animal model of autosomal recessive RP are in temporal association with the process of photoreceptor demise and that *in vivo* inhibition of *de novo* ceramide biosynthesis delays effectively this process (Strettoi et al., 2010; Piano et al., 2013). This observation supports the notion that this sphingolipid is involved in the neurodegenerative process taking place in RP. The results are in agreement with the increased ceramide levels found in tissues from individuals with other pathologies leading to apoptosis, such as brain tissue from Alzheimer’s disease patients and thus provide additional evidence that elevated ceramide is a common pathogenic factor of a variety of neurodegenerative diseases (Han et al., 2002; He et al., 2009; Kurek et al., 2017). Both *in vitro* and *in vivo* studies on various animal models confirm that ceramide is involved in processes that trigger apoptotic death of photoreceptors and that treatments aiming at to reducing ceramide content are beneficial to cell viability and function (Acharya, 2003; Acharya and Acharya, 2005; German et al., 2006; Sanvicens and Cotter, 2006; Rotstein et al., 2010).

The present study suggests that Myriocin, a selective inhibitor of SPT, the rate-limiting enzyme of ceramide biosynthesis, exerts rescue effects on the (rather acute) retinal degeneration process occurring in Tvrm4 mice, carrying a dominant mutation of rhodopsin inducible by light in adulthood and therefore completely different from the rd10 mutants previously employed.

We show that, similarly to rd10 mice, rescue effects obtained with a single intraocular injection of 1.88 mM Myriocin reduce the number of pycnotic photoreceptors, but this is not sufficient to efficiently rescue the ERG response (Strettoi et al., 2010) and does not change reproducibly ceramide levels (data not shown). Increasing the dose of Myriocin to 10 mM results in a similarly limited recovery of visual function and reveals single-case correlations between ERG amplitude and levels of retinal ceramide, without reducing it significantly on averaged data. WB analysis shows a Myriocin-related tendency to restore the levels of enzymes involved in apoptotic processes (caspase 3) and oxidative stress (Sod1) toward physiological values, indicating a reduction in the degenerative process. Most likely, the lack of functional effects after intravitreal treatment with Myriocin depends on the short duration of the treatment that fails to save a number of photoreceptors large enough to affect a mass-recording measurement such as the ERG. Moreover, the intrinsic variability in the size of the light-induced, degenerating area typical of the Tvrm4 phenotype makes the acutely induced and treated model quite complex for quantitative mass assays, despite the fact that we invariably use the contralateral, vehicle-treated eyes as controls. Conversely, a measurable decrement in the number of degenerating photoreceptor profiles (which are counted with single-cell resolution) is achieved with a single i.v. injection of 1.88 mM Myriocin, demonstrating efficacy in the acute phase of degeneration as already shown on the rd10 model (Strettoi et al., 2010).

To extend the time window of Myriocin treatment and appreciate the likely beneficial effects on the Tvrm4 retinal phenotype, we chose sustained (5-day) treatment achieved by the systemic administration of the drug by intraperitoneal injections (Osuchowski et al., 2004). Intraperitoneal treatment did not cause any adverse effect at general and ocular level although specific toxicity studies will be necessary in the future. Indeed, sustained, i.p. administration of Myriocin in Tvrm4 mice effectively reduces the size of the central-degenerating retina, rescues rhodopsin levels, and preserves the scotopic ERG. These results strongly suggest that Myriocin, or some active, yet unknown metabolite, are able to permeate the blood–retinal barrier, which, in these animals, could already be damaged due to the degenerative processes in progress and reduce the processes that lead to the death of photoreceptors. Indeed, levels of activated caspase-3 protein are reduced as well. Our experiment indications about levels of physiological defense pathways against the oxidative stress implicated in secondary cone degeneration (Usui et al., 2011) show that Myriocin i.p. treatment is associated with a significant increase in the content of the cytosolic isoform of retinal Sod1. Hence, Myriocin is not only an effective anti-apoptotic agent acting by inhibiting SPT and decreasing ceramide levels, in turn involved also in ROS generation (Sanvicens and Cotter, 2006) but is also capable of increasing the physiological anti-oxidant defenses by stimulating the synthesis of the key enzyme Sod1.

Although the efficacy of Myriocin as an anti-apoptotic and as a regulator of the cell cycle has already been proven in other tissues by means of either local (Reforgiato et al., 2016) and systemic administration to target cancer cells (Lee et al., 2012) or lipid metabolism in an animal model of type I diabetes (Kurek et al., 2017), our study is the first that demonstrates the ability of Myriocin, or of some active metabolite, to act effectively on the retina, an outpost of the brain, even when administered systemically, exerting a measurable anti-degenerative action and activating local anti-oxidant defenses. An anti-inflammatory benefit on retinal survival is not to be excluded, given the proven action of Myriocin as an immunosuppressant and anti-inflammatory agent (Caretti et al., 2014) and the increasing evidence of a contribution of inflammation-immune response on worsening of retinal phenotype in inherited photoreceptor degeneration (Zabel et al., 2016; Guadagni et al., 2019).

## Data Availability Statement

The datasets generated for this study are available on request to the corresponding author.

## Ethics Statement

The animal study was reviewed and approved by Italian Ministry of Health and Ethical Committees of Department of Pharmacy, University of Pisa and CNR Neuroscience Institute.

## Author Contributions

IP, VD’A, and EN conceived and performed the experiments. MB contributed to the execution of the experiments. MD and RP performed the experiments on ceramide analysis. IP, VD’A, CG, and ES collected and critically analyzed the data. IP and CG wrote the manuscript. RG and ES critically reviewed the manuscript. All authors read, edited, and approved the final manuscript.

## Conflict of Interest

RG, ES, and CG have a patent for the employment of inhibitors of serine palmitoyltransferase for preventing and delaying retinitis pigmentosa (World Patent PCT/EP2010001119).

The remaining authors declare that the research was conducted in the absence of any commercial or financial relationships that could be construed as a potential conflict of interest.

## Footnotes

1 http://www.sph.uth.tmc.edu/RetNet/

## References

[B1] Acharya N. K. Qi X. Goldwaser E. L. Godsey G. A. Wu H. Kosciuk M. C. (2017). Retinal pathology is associated with increased blood–retina barrier permeability in a diabetic and hypercholesterolaemic pig model: beneficial effects of the LpPLA _2_ inhibitor darapladib. *Diab. Vasc. Dis. Res.* 14 200–213. 10.1177/1479164116683149 28301218

[B2] Acharya U. (2003). Modulating sphingolipid biosynthetic pathway rescues photoreceptor degeneration. *Science* 299 1740–1743. 10.1126/science.1080549 12637747

[B3] Acharya U. Acharya J. K. (2005). Enzymes of sphingolipid metabolism in *Drosophila melanogaster*. *CMLS Cell. Mol. Life Sci.* 62 128–142. 10.1007/s00018-004-4254-1 15666085 PMC11924467

[B4] Athanasiou D. Aguila M. Bellingham J. Li W. McCulley C. Reeves P. J. (2018). The molecular and cellular basis of rhodopsin retinitis pigmentosa reveals potential strategies for therapy. *Prog. Retin. Eye Res.* 62 1–23. 10.1016/j.preteyeres.2017.10.002 29042326 PMC5779616

[B5] Barone I. Novelli E. Piano I. Gargini C. Strettoi E. (2012). Environmental enrichment extends photoreceptor survival and visual function in a mouse model of retinitis pigmentosa. *PLoS One* 7:e50726. 10.1371/journal.pone.0050726 23209820 PMC3508993

[B6] Budzynski E. Gross A. K. McAlear S. D. Peachey N. S. Shukla M. He F. (2010). Mutations of the opsin gene (Y102H and I307N) lead to light-induced degeneration of photoreceptors and constitutive activation of phototransduction in mice. *J. Biol. Chem.* 285 14521–14533. 10.1074/jbc.M110.112409 20207741 PMC2863193

[B7] Caretti A. Bragonzi A. Facchini M. De Fino I. Riva C. Gasco P. (2014). Anti-inflammatory action of lipid nanocarrier-delivered myriocin: therapeutic potential in cystic fibrosis. *Biochim. Biophys. Acta* 1840 586–594. 10.1016/j.bbagen.2013.10.018 24141140 PMC4097882

[B8] Chang B. Hawes N. L. Hurd R. E. Davisson M. T. Nusinowitz S. Heckenlively J. R. (2002). Retinal degeneration Mutants in the Mouse. *Vis. Res.* 42 517–525. 10.1016/S0042-6989(01)00146-8 11853768

[B9] Garanto A. Mandal N. A. Egido-Gabás M. Marfany G. Fabriàs G. Anderson R. E. (2013). Specific sphingolipid content decrease in cerkl knockdown mouse retinas. *Exp. Eye Res.* 110 96–106. 10.1016/j.exer.2013.03.003 23501591 PMC4019014

[B10] Gargini C. Novelli E. Piano I. Biagioni M. Strettoi E. (2017). Pattern of retinal morphological and functional decay in a light-inducible, rhodopsin mutant mouse. *Sci. Rep.* 7:5730. 10.1038/s41598-017-06045-x 28720880 PMC5516022

[B11] Gargini C. Terzibasi E. Mazzoni F. Strettoi E. (2007). Retinal organization in the retinal degeneration 10 (Rd10) mutant mouse: a morphological and ERG study. *J. Comp. Neurol.* 500 222–238. 10.1002/cne.21144 17111372 PMC2590657

[B12] German O. L. Miranda G. E. Abrahan C. E. Rotstein N. P. (2006). Ceramide is a mediator of apoptosis in retina photoreceptors. *Invest. Opthalmol. Vis. Sci.* 47 1658. 10.1167/iovs.05-1310 16565407

[B13] Glaros E. N. Kim W. S. Wu B. J. Suarna C. Quinn C. M. Rye K. A. (2007). Inhibition of atherosclerosis by the serine palmitoyl transferase inhibitor myriocin is associated with reduced plasma glycosphingolipid concentration. *Biochem. Pharmacol.* 73 1340–1346. 10.1016/j.bcp.2006.12.023 17239824

[B14] Glaros E. N. Kim W. S. Garner B. (2010). Myriocin-mediated up-regulation of hepatocyte apoA-I synthesis is associated with ERK inhibition. *Clin. Sci.* 118 727–736. 10.1042/CS20090452 20102334 PMC2860698

[B15] Guadagni V. Biagioni M. Novelli E. Aretini P. Mazzanti C. M. Strettoi E. (2019). Rescuing cones and daylight vision in retinitis pigmentosa mice. *FASEB J.* 33 10177–10192. 10.1096/fj.201900414R 31199887 PMC6764477

[B16] Gürtler A. Kunz N. Gomolka M. Hornhardt S. Friedl A. A. McDonald K. (2013). Stain-free technology as a normalization tool in western blot analysis. *Anal. Biochem.* 433 105–111. 10.1016/j.ab.2012.10.010 23085117

[B17] Han X. Holtzman M. D. McKeel D. W. Jr. Kelley J. Morris J. C. (2002). Substantial sulfatide deficiency and ceramide elevation in very early Alzheimer’s disease: potential role in disease pathogenesis: sulfatide deficiency and ceramide elevation in AD. *J. Neurochem.* 82 809–818. 10.1046/j.1471-4159.2002.00997.x 12358786

[B18] Hancock H. A. Kraft T. W. (2004). Oscillatory potential analysis and ERGs of normal and diabetic rats. *Invest. Opthalmol. Vis. Sci.* 45:1002. 10.1167/iovs.03-1080 14985323

[B19] He B. Lu N. Zhou Z. (2009). Cellular and nuclear degradation during apoptosis. *Curr. Opin. Cell Biol.* 21 900–912. 10.1016/j.ceb.2009.08.008 19781927 PMC2787732

[B20] Kurek K. Garbowska M. Ziembicka D. M. Łukaszuk B. Rogowski J. Chabowski A. (2017). Myriocin treatment affects lipid metabolism in skeletal muscles of rats with streptozotocin-induced type 1 diabetes. *Adv. Med. Sci.* 62 65–73. 10.1016/j.advms.2016.04.003 28189121

[B21] Lamb T. D. Pugh E. N. Jr. (1992). A quantitative account of the activation steps involved in phototransduction in amphibian photoreceptors. *J. Physiol.* 449 719–758. 10.1113/jphysiol.1992.sp019111 1326052 PMC1176104

[B22] Lee Y. S. Choi K. M. Lee S. Sin D. M. Lim Y. Lee Y. M. (2012). Myriocin, a serine palmitoyltransferase inhibitor, suppresses tumor growth in a murine melanoma model by inhibiting de novo sphingolipid synthesis. *Cancer Biol. Ther.* 13 92–100. 10.4161/cbt.13.2.18870 22336910

[B23] Lei B. Yao G. Zhang K. Hofeldt K. J. Chang B. (2006). Study of rod- and cone-driven oscillatory potentials in mice. *Invest. Opthalmol. Vis. Sci.* 47:2732. 10.1167/iovs.05-1461 16723493

[B24] Lin J. H. Li H. Yasumura D. Cohen H. R. Zhang C. Panning B. (2007). IRE1 signaling affects cell fate during the unfolded protein response. *Science* 318 944–949. 10.1126/science.1146361 17991856 PMC3670588

[B25] MacLaren R. E. Bennett J. Schwartz S. D. (2016). Gene therapy and stem cell transplantation in retinal disease: the new frontier. *Ophthalmology* 123 S98–S106. 10.1016/j.ophtha.2016.06.041 27664291 PMC5545086

[B26] Mendes H. F. Cheetham M. E. (2008). Pharmacological manipulation of gain-of-function and dominant-negative mechanisms in rhodopsin retinitis pigmentosa. *Hum. Mol. Genet.* 17 3043–3054. 10.1093/hmg/ddn202 18635576

[B27] Mendez A. Lem J. Simon M. Chen J. (2003). Light-dependent translocation of arrestin in the absence of rhodopsin phosphorylation and transducin signaling. *J. Neurosci.* 23 3124–3129. 10.1523/JNEUROSCI.23-08-03124.2003 12716919 PMC6742335

[B28] Osuchowski M. F. Johnson V. J. He Q. Sharma R. P. (2004). Myriocin, a serine palmitoyltransferase inhibitor, alters regional brain neurotransmitter levels without concurrent inhibition of the brain sphingolipid biosynthesis in mice. *Toxicol. Lett.* 147 87–94. 10.1016/j.toxlet.2003.10.016 14700532

[B29] Piano I. D’Antongiovanni V. Testai L. Calderone V. Gargini C. (2019). A nutraceutical strategy to slowing down the progression of cone death in an animal model of retinitis pigmentosa. *Front. Neurosci.* 13:461. 10.3389/fnins.2019.00461 31156364 PMC6533548

[B30] Piano I. Novelli E. Santina L. D. Strettoi E. Cervetto L. Gargini C. (2016). Involvement of autophagic pathway in the progression of retinal degeneration in a mouse model of diabetes. *Front. Cell. Neurosci.* 10:42. 10.3389/fncel.2016.00042 26924963 PMC4759287

[B31] Piano I. Novelli E. Gasco P. Ghidoni R. Strettoi E. Gargini C. (2013). Cone survival and preservation of visual acuity in an animal model of retinal degeneration. *Eur. J. Neurosci.* 37 1853–1862. 10.1111/ejn.12196 23551187

[B32] Platania C. B. M. Cas M. D. Cianciolo S. Fidilio A. Lazzara F. Paroni R. (2019). Novel ophthalmic formulation of myriocin: implications in retinitis pigmentosa. *Drug Deliv.* 26 237–243. 10.1080/10717544.2019.1574936 30883241 PMC6419690

[B33] Ranty M. L. Carpentier S. Cournot M. Rico-Lattes I. Malecaze F. Levade T. (2009). Ceramide production associated with retinal apoptosis after retinal detachment. *Graefes Arch. Clin. Exp. Ophthalmol.* 247 215–224. 10.1007/s00417-008-0957-6 18958490

[B34] Reforgiato M. R. Milano G. Fabriàs G. Casas J. Gasco P. Paroni R. (2016). Inhibition of ceramide de novo synthesis as a postischemic strategy to reduce myocardial reperfusion injury. *Basic Res. Cardiol.* 111:12. 10.1007/s00395-016-0533-x 26786259

[B35] Rotstein N. P. Miranda G. E. Abrahan C. E. German O. L. (2010). Regulating survival and development in the retina: key roles for simple sphingolipids. *J. Lipid Res.* 51 1247–1262. 10.1194/jlr.R003442 20100817 PMC3035489

[B36] Sanvicens N. Cotter T. G. (2006). Ceramide is the key mediator of oxidative stress-induced apoptosis in retinal photoreceptor cells. *J. Neurochem.* 98 1432–1444. 10.1111/j.1471-4159.2006.03977.x 16923157

[B37] Stefanov A. Novelli E. Strettoi E. (2019). Inner retinal preservation in the photoinducible I307N rhodopsin mutant mouse, a model of autosomal dominant retinitis pigmentosa. *J. Comp. Neurol.* 528 1502–1522. 10.1002/cne.24838 31811649 PMC7187456

[B38] Strettoi E. Gargini C. Novelli E. Sala G. Piano I. Gasco P. (2010). Inhibition of ceramide biosynthesis preserves photoreceptor structure and function in a mouse model of retinitis pigmentosa. *Proc. Natl. Acad. Sci. U.S.A.* 107 18706–18711. 10.1073/pnas.1007644107 20937879 PMC2972949

[B39] Sung C. H. Davenport C. M. Nathans J. (1993). Rhodopsin mutations responsible for autosomal dominant retinitis pigmentosa. Clustering of functional classes along the polypeptide chain. *J. Biol. Chem.* 268 26645–26649. 8253795

[B40] Sung C. H. Schneider B. G. Agarwal N. Papermaster D. S. Nathans J. (1991). Functional heterogeneity of mutant rhodopsins responsible for autosomal dominant retinitis pigmentosa. *Proc. Natl. Acad. Sci. U.S.A.* 88 8840–8844. 10.1073/pnas.88.19.8840 1924344 PMC52606

[B41] Tam B. M. Moritz O. L. (2009). The role of rhodopsin glycosylation in protein folding, trafficking, and light-sensitive retinal degeneration. *J. Neurosci.* 29 15145–15154. 10.1523/JNEUROSCI.4259-09.2009 19955366 PMC6665958

[B42] Tuson M. Marfany G. Gonzàlez-Duarte R. (2004). Mutation of CERKL, a novel human ceramide kinase gene, causes autosomal recessive retinitis pigmentosa (RP26). *Am. J. Hum. Genet.* 74 128–138. 10.1086/381055 14681825 PMC1181900

[B43] Usui S. Oveson B. C. Iwase T. Lu L. Lee S. Y. Jo Y. J. (2011). Overexpression of SOD in retina: need for increase in H2O2-detoxifying enzyme in same cellular compartment. *Free Radic. Biol. Med.* 51 1347–1354. 10.1016/j.freeradbiomed.2011.06.010 21736939 PMC3163708

[B44] Wright A. F. Chakarova C. F. Abd El-Aziz M. M. Bhattacharya S. S. (2010). Photoreceptor degeneration: genetic and mechanistic dissection of a complex trait. *Nat. Rev. Genet.* 11 273–284. 10.1038/nrg2717 20212494

[B45] Zabel M. K. Zhao L. Zhang Y. Gonzalez S. R. Ma W. Wang X. (2016). Microglial phagocytosis and activation underlying photoreceptor degeneration is regulated by CX3CL1-CX3CR1 signaling in a mouse model of retinitis pigmentosa: CX3CR1 signaling in retinal degeneration. *Glia* 64 1479–1491. 10.1002/glia.23016 27314452 PMC4958518

